# Reducing age bias in decision analyses of anticoagulation for patients with nonvalvular atrial fibrillation – A microsimulation study

**DOI:** 10.1371/journal.pone.0199593

**Published:** 2018-07-11

**Authors:** Matthew A. Pappas, Sandeep Vijan, Michael B. Rothberg, Daniel E. Singer

**Affiliations:** 1 Center for Value-Based Care Research, Medicine Institute, Cleveland Clinic, Cleveland, Ohio, United States of America; 2 Division of General Internal Medicine, Department of Internal Medicine, The University of Michigan Health System, Ann Arbor, Michigan, United States of America; 3 Division of General Internal Medicine, Massachusetts General Hospital and Harvard Medical School, Boston, Massachusetts, United States of America; Hospital Clinico San Carlos, SPAIN

## Abstract

**Background:**

Anticoagulation decreases a patient’s risk of ischemic stroke and increases the risk of hemorrhage. Decision analyses regarding anticoagulation therefore require that different outcomes be weighted in comparison to one another. Most decision analyses to date have weighted intracranial hemorrhage (ICH) as 1.5 times worse than ischemic stroke, but because death and disability have lifelong impact, the expected impact should vary by life expectancy. Therefore, a fixed weighting ratio leads to age-related bias decision analyses of anticoagulation. We aimed to quantify the relative impact of ICH and ischemic stroke and derive a ratio that allows decision analysis without microsimulation.

**Methods:**

We created a microsimulation model to predict QALYs lost due to ICH and ischemic stroke. We then applied a meta-model to predict the ratio of QALYs lost from ICH relative to ischemic stroke.

**Results:**

Previously-used weighting ratios (1.5) are close to our derived mean weighting ratio (1.60). However, the weighting ratio of QALYs lost from ICH relative to ischemic stroke is sensitive to age and discount rate. Patients at younger ages have higher mean weighting ratios, as do patients with higher discount rates.

**Conclusions:**

The ratio of QALYs lost to ICH relative to ischemic stroke varies with age and discount rate. We present a set of such ratios here for use in decision analyses that do not incorporate full microsimulation models. Use of weighting ratios that vary with age, rather than the current fixed ratios, has the potential to reduce age-based bias in decision-making regarding events with lifelong implications. In this case, use of dynamic ratios may change anticoagulation recommendations for patients with nonvalvular atrial fibrillation at relatively low stroke risk.

## Introduction

Anticoagulation decreases a patient’s risk of ischemic stroke, while increasing the risk of hemorrhage. Decision analyses regarding anticoagulation therefore require comparing the harm caused by ischemic stroke and hemorrhagic complications. In recognition of the generally worse outcomes of intracranial hemorrhage (ICH) compared with ischemic stroke, many estimates of net clinical benefit and cost-effectiveness analyses use a relative weight of 1.5, with sensitivity analyses from 1.0 to 2.0.[1–14] Other authors have used similar methods, with different weighting schema.[15,16] Results of those analyses have influenced current guidelines.[17–19]

This weighting of different outcomes, though, carries inherent limitations. First, the appropriate weight–how much more severe the outcomes of ICH are relative to the outcomes of ischemic stroke–is unclear. More importantly, the use of a uniform weight across different ages and risk factors may mask patient heterogeneity. The relative impact of ICH and ischemic stroke are dependent on other patient-specific factors, most notably life expectancy. It may therefore be appropriate to use different weights for different subpopulations, any of which may differ meaningfully from the fixed weights that are currently applied.

To illustrate the expected relationship, imagine a patient with a remaining life expectancy of one week. Any event that leads to a one-week hospitalization will have a similar impact on remaining quality-adjusted life years (QALYs). The ratio of QALYs lost from an ICH to QALYs lost from an ischemic stroke would approach one in such a patient. By contrast, a patient with decades of life expectancy remaining will experience a loss of QALYs that may be very different for different events. Depending on the mortality and long-term disability of adverse events, the ratio between events compared may diverge considerably from one.

Despite these inherent limitations, a ratio weighting the expected outcomes of ICH and ischemic stroke remains valuable. Many agents, from antiplatelet agents to anticoagulants to thrombolytic agents, have similar trade-offs and are used for many different indications. A decision analysis weighting principal outcomes requires less methodologic expertise than a full microsimulation, and thus makes possible more carefully analyzed decision-making for a wider range of medications and indications.

We therefore set out to derive the ratio of QALYs lost to ICH compared with ischemic stroke among patients with nonvalvular atrial fibrillation, to be used in future decision-analytic models.

## Methods

**Fig 1:**
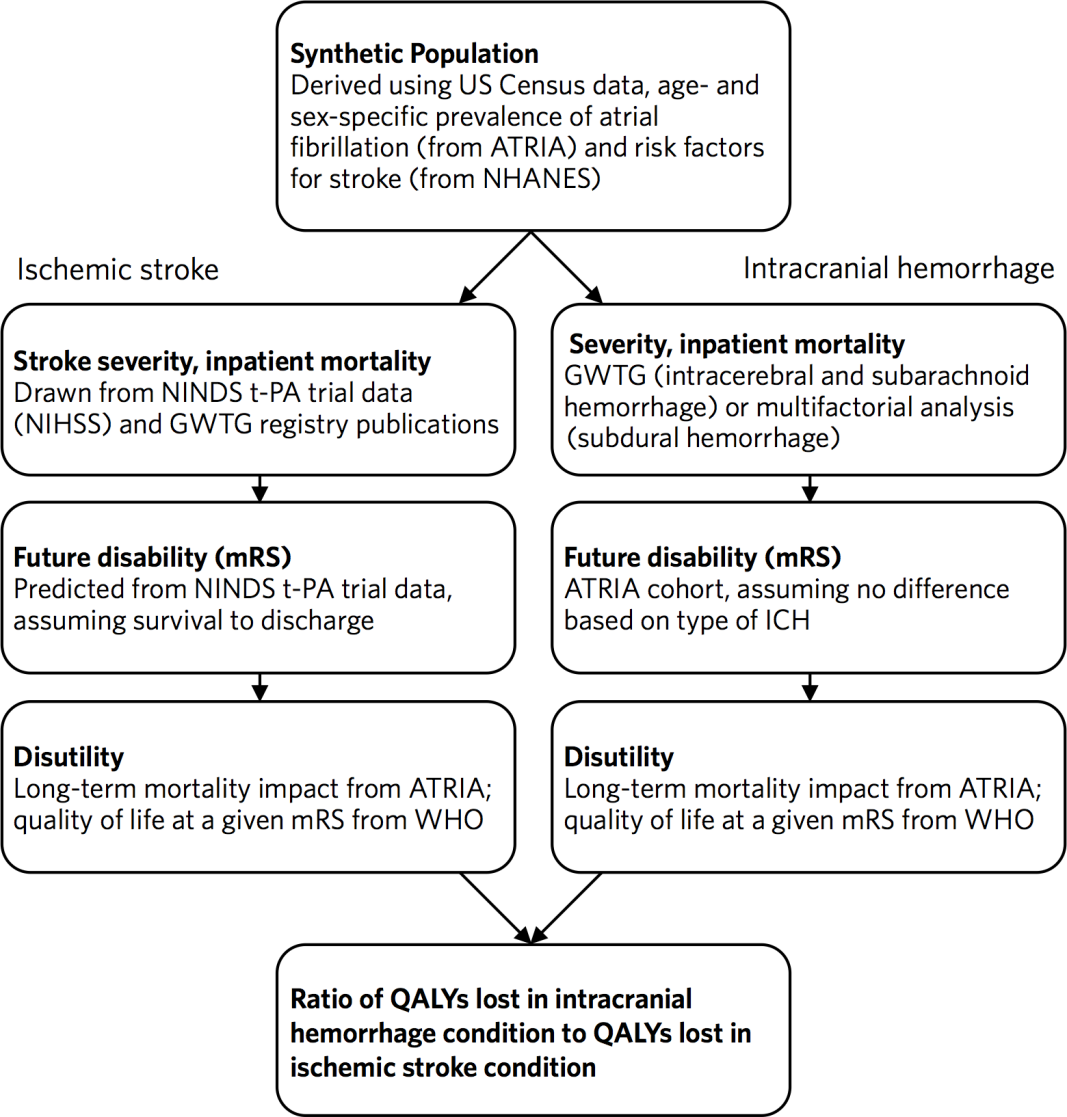
Schematic diagram of microsimulation model.

We designed a Monte Carlo simulation predicting the QALYs lost to ICH compared to ischemic stroke.[20] We began with a synthetic population intended to mirror the atrial fibrillation population of the United States. Each hypothetical patient was simulated in an ischemic stroke condition and an ICH condition, drawing from a variety of datasets to predict downstream morbidity and mortality. The QALYs lost in each condition, and the ratio of QALYs lost in each of the two conditions, were calculated. We then created a regression model of the simulation results (a “meta-model”) to demonstrate the influence of the input variables on this ratio, and predicted the marginal QALY loss ratio at various ages. A schematic diagram of our model can be found in Fig 1, and a summary of our model inputs can be found in Table 1. Additional description of our model can be found in the S1 Appendix. All analyses were performed in version 13 of Stata (College Station, TX).

**Table 1:** Sources of estimates used to build simulation model.

Modeled variable	Mean (Median)	sd (IQR)	Distribution	Reference(s)
Age and sex of US population	N/A	N/A	N/A	[21]
Age- and sex-specific prevalence of atrial fibrillation	N/A	N/A	N/A	[22]
Age- and sex-specific prevalence and covariation of stroke risk factors	N/A	N/A	N/A	[31]
Ischemic stroke severity, NIHSS	16.2	7.0	Normal	[24,25]
Percentage of intracranial hemorrhages (ICH) that are intracerebral	65.2%	-	Fixed	[23]
Percentage of ICH that are subarachnoid	5.8%	-	Fixed	[23]
Percentage of ICH that are subdural	29.0%	-	Fixed	[23]
Severity of intracerebral hemorrhages (NIHSS)	(9)	(3–19)	Gamma	[24]
Severity of subarachnoid hemorrhages (NIHSS)	(3)	(0–11)	Gamma	[24]
Inpatient mortality, ischemic stroke	Predicted	N/A	N/A	[25]
Inpatient mortality, intracerebral and subarachnoid hemorrhages	Predicted	N/A	N/A	[25]
Inpatient mortality, subdural hemorrhages	Predicted	N/A	N/A	[26]
Future modified Rankin Score (mRS) following ischemic stroke	Predicted, see S1 Appendix	N/A	N/A	[27]
Future mRS following ICH, assuming survival to discharge	13.8% each mRS 0–2, 19.5% each mRS 3–5	N/A	N/A	[28]
Length of stay, conditioned on diagnosis	Sampled	N/A	N/A	[35]
Hazard ratio for long-term mortality following event, mRS < = 2	1.7	-	Fixed	[29]
Hazard ratio for long-term mortality following event, mRS = 3 or 4	2.9	-	Fixed	[29]
Hazard ratio for long-term mortality following event, mRS 5	8.3	-	Fixed	[29]
Baseline probabilities of death by age	Varies	N/A	N/A	[30]
Discount rate	3%	1.7%	Uniform, 0 to 6%	Assumed

### Synthetic population

Our population was modeled on the most recent year of the National Health and Nutrition Examination Survey (NHANES) for which risk factors of stroke and in-hospital mortality following stroke are available (2011–2012).[31] Because atrial fibrillation is not included in NHANES, this diagnosis was added separately, using age-specific prevalence of atrial fibrillation in the Anticoagulation and Risk Factors in Atrial Fibrillation (ATRIA) cohort.[22] Using those data and the US Census estimates, we created a synthetic population intended to mirror the size, age distribution, and risk factors of the US atrial fibrillation population.[21]

### Stroke severity and mortality

Decision analyses in anticoagulation estimate the impact of ICH for patients on anticoagulation relative to the impact of ischemic stroke without anticoagulation. Therefore, event severity and outcomes used must reflect those two states (ICH with anticoagulation and ischemic stroke without anticoagulation). In the ischemic stroke condition, we drew ischemic stroke severity (as measured by the NIH Stroke Scale, NIHSS) from the subset of patients enrolled in the NINDS t-PA trial who had atrial fibrillation, using a bootstrapping approach.[27] Because warfarin use was an exclusion criterion of the trial, we can be confident that this sample includes only patients with known atrial fibrillation who were not anticoagulated. We then calculated the probability of in-hospital mortality using a previously-published logistic regression model. [24,25]

We designated each ICH as intracerebral, subarachnoid, or subdural, randomly and in keeping with the proportions observed among the combined warfarin groups of RE-LY and ROCKET-AF.[23] We did not consider epidural hemorrhages. For patients who sustained intracerebral hemorrhages, we assigned an NIHSS using a normal distribution based on the median and interquartile range (IQR) observed in the Get With the Guidelines-Stroke (GWTG) registry, a large dataset that collects abstracted data from over 1,000 participating hospitals.[24] For patients who sustained subarachnoid hemorrhages, we assigned an NIHSS using a gamma distribution fitted to the median and IQR observed in the same registry. For each type of event, we calculated the probability of in-hospital mortality using a previously-published logistic regression model from the same GWTG-Stroke registry, which appeared to have excellent discrimination in split-sample validation (c-statistics of 0.82–0.89).[25] While patient characteristics (such as age and sex) and comorbidities (such as diagnoses of diabetes, coronary artery disease, and prior stroke) were included in our synthetic population, other predictors, such as presentation via ambulance and time of arrival to the Emergency Department, required other assumptions as detailed in the S1 Appendix.) For hypothetical patients who sustained subdural hemorrhages, we used a previously-published multifactorial analysis.[26] Interestingly, neither warfarin use nor coagulopathy are included as predictors of mortality in the GWTG-Stroke publications addressing ischemic stroke, intracerebral hemorrhage, and subarachnoid hemorrhage, though both (warfarin use and coagulopathy) are included in the multifactorial analysis we used to estimate subdural hemorrhage mortality.

### Future disability

For hypothetical patients who survive to hospital discharge, we then predicted modified Rankin Scores (mRS) 3 months following the simulated event. In the ischemic stroke condition, we used an ordered logistic regression derived from NINDS t-PA trial data.[27] In the ICH condition, we followed the rates of disability published by the ATRIA cohort, assuming that “minor disability” was evenly distributed between mRS of 1 and 2, that “major disability” was evenly distributed among mRS of 3–5, and assuming no differences in rates of disability based on type of ICH.[28]

### Disutilities

To estimate the disutility of hospitalization, we drew length-of-stay from the National Inpatient Sample (NIS), conditioned on principal diagnosis and use of thrombolytics (a randomly assigned 10% of patients in the ischemic stroke condition). We estimated the disutility of the hospitalization as a function of length of stay (see S1 Appendix for further detail). [32,33]

For patients who survive to discharge, we calculated life expectancy using published life tables and applying mRS-specific hazard ratios observed in post-ischemic stroke patients.[29,30] We then calculated remaining QALYs, conditioned on mRS, discounted to the present, and calculated the QALYs lost relative to baseline.

### Ratio and meta-model

We then divided each hypothetical patient’s QALYs lost in the ICH condition by the QALYs lost in the ischemic stroke condition, to yield a ratio of the impact of ICH relative to ischemic stroke. We then created a regression model of that ratio (“meta-model”), using patient-specific input variables (age, congestive heart failure, hypertension, diabetes, prior stroke, coronary artery disease, dyslipidemia, weight, and discount rate) as predictors. Because any predictor is likely to be statistically significant in a large simulation sample (here over 3 million hypothetical patients), we removed predictors from the meta-model if varying the input variable from the 5^th^ to the 95^th^ percentile did not change the predicted ratio by more than 10%. This left only age and discount rate in our meta-model. We then tested for nonlinear relationships.

### Sensitivity analyses

By using a meta-model, we tested the sensitivity of our primary outcome to age, congestive heart failure, hypertension, diabetes, prior stroke, coronary artery disease, dyslipidemia, weight, and discount rate.

### Reclassification testing

To assess whether use of a variable weight would lead to changes in treatment recommendation, we recalculated net clinical benefit from a prior analysis, using variable weights rather than previously-used fixed weights.[1] We noted, for each CHADS_2_ score, groups whose mean predicted benefit changed from positive to negative net clinical benefit (harm) over the range of predicted weights in our final meta-model.

## Results

**Fig 2:**
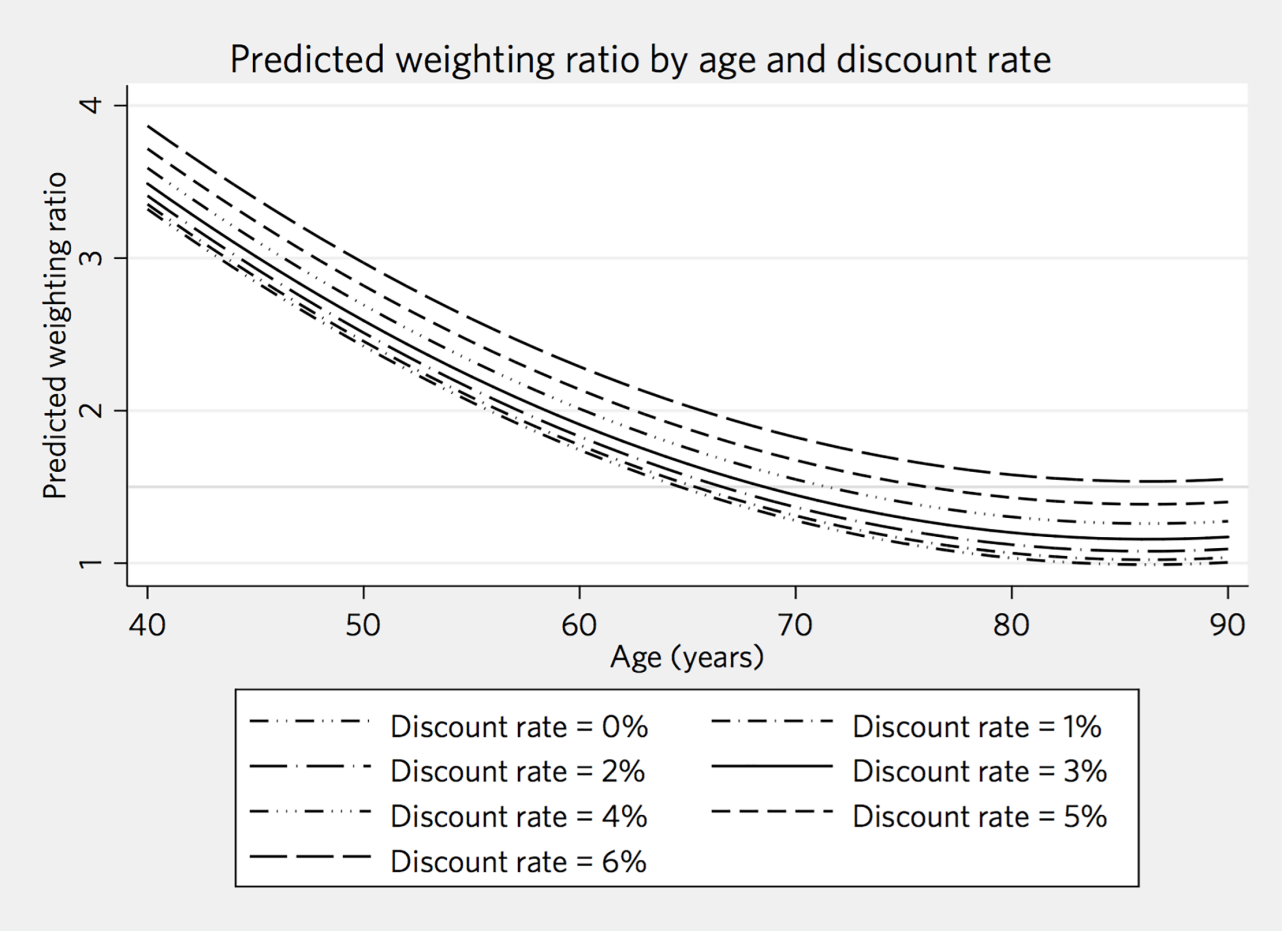
Predicted marginal weighting ratio, as a function of age.

The mean QALY impact of ICH relative to that of ischemic stroke was 1.60 (median 1.03, IQR 0.71–1.85). In our meta-model, age and discount rate are significant predictors of the weighting ratio, and each had nonlinear effects on the predicted ratio. An interaction between age and discount rate did not improve model fit. Younger patients have, on average, a higher ratio of QALYs lost from ICH compared with ischemic stroke. Similarly, higher discount rates lead to higher predicted ratios. The results of our final meta-model are shown in Table 2, the marginal predicted weighting ratio at each decade of life is shown at different ages in Table 3, and a plot of the predicted marginal weighting ratio, as a function of age, is shown in Fig 2. Because they drive our results, we have included selected intermediate results (inpatient mortality and downstream disability) in Tables 4 and 5.

**Table 2:** Results of final meta-model.

β_(age × age)_	0.001087
β_age_	-0.1876288
β_(discount_rate × discount_rate)_	117.5787
β_(discount_rate)_	2.046664
β_0_	9.086773

All coefficients are highly statistically significant (p<0.001). R2 for final model ≅ 0.14; n ≅ 3.03 million.

**Table 3:** Predicted marginal ratio of QALYs lost from ICH, relative to ischemic stroke, at selected ages and discount rates.

		Discount rate		
		**2%**	**4%**	**6%**
**Age**	**40**	3.41	3.59	3.87
	**50**	2.51	2.69	2.97
	**60**	1.83	2.01	2.29
	**70**	1.37	1.55	1.83
	**80**	1.12	1.30	1.58
	**90**	1.09	1.27	1.55

**Table 4:** Intermediate results: In-hospital mortality, by event.

Intermediate outcome	Mean
Mortality, ischemic stroke	13.6%
Mortality, intracranial hemorrhage	26.0%
– Mortality, intracerebral hemorrhage	22.9%
– Mortality, subarachnoid hemorrhage	22.1%
– Mortality, subdural hemorrhage	33.7%

n.b.: Variance is fixed, due to the dichotomous measure.

**Table 5:** Intermediate results: Disability 3-months following discharge, conditional on survival to hospital discharge and stratified by event.

**Modified Rankin Score (mRS)**	**0**	**1**	**2**	**3**	**4**	**5**	**6**
Ischemic stroke	0.7%	13.9%	25.0%	29.0%	22.0%	8.7%	0.8%
Intracranial hemorrhage	13.8%	13.8%	13.8%	19.6%	19.6%	19.5%	0

n.b.: All subsets of intracranial hemorrhage are assumed to have equal post-discharge disability.

Use of weights that varied over our marginal predicted range led to reclassification from benefit to harm in patients with a CHADS_2_ score of 1. While the magnitude of predicted net clinical benefit (or harm) changed for other groups, the mean did not change from benefit to harm over the range of our marginal predicted weights.

## Discussion

Intracranial hemorrhages lead to generally worse outcomes relative to ischemic strokes. To estimate how much worse, prior decision analyses and cost-effectiveness analyses in anticoagulation have assumed ICH to be 1.5 times worse than ischemic stroke. In this modeling study, we used a microsimulation model to derive a ratio of QALYs lost to each outcome, to better inform future decision analyses. We found that the mean relative ratio of QALYs lost to ICH relative to ischemic stroke is close to the usual base-case estimate (an overall population mean of 1.60, in our analysis, compared to 1.5 in most prior work).

More importantly, we demonstrated that the appropriate weighting ratio varies by age, with lower ratios for older patients and higher ones for younger patients. Using a fixed ratio across the spectrum of age has led previous decision-analytic models to overvalue anticoagulation in the young and undervalue anticoagulation in the elderly. This is consistent with our hypothesis; indeed, the relative impact of any two events that induce different rates of mortality and long-term disability should vary by life expectancy, and age is a strongly related to life expectancy. Because atrial fibrillation is in large part a disease of aging, age-related biases could lead to important shortcomings in who is recommended for treatment.

If adopted, this has the potential to change which patients would be recommended for anticoagulation. For example, in a prior analysis stratifying net clinical benefit by CHADS_2_ score, patients with a CHADS_2_ score of 1 would benefit, on average, from anticoagulation (though with a confidence interval overlapping zero).[1] Using an age-varying weight and a discount rate of 3%, a 50-year-old patient (our derived weight: 2.59) whose CHADS_2_ score is 1 would move from predicted net clinical benefit using the standard weight to net harm. Conversely, using a variable weight would increase the predicted net clinical benefit for a 90-year-old patient with a CHADS_2_ score of 1 from 0.19 to 0.27. Guidelines incorporating varying weights would recommend against anticoagulation for patients with a CHADS_2_ of 1 at age 50 and in favor at age 90 (although, of course, competing risks and other disutilities could change such a recommendation). While use of variable weights would be unlikely to change recommendations for patients at high or intermediate stroke risk, large numbers of patients currently recommended for anticoagulation are at low stroke risk.[34] Those low-risk patients may be reclassified using these estimates.

Our analysis is subject to a number of important limitations. First, this method is only useful insofar as decision analyses continue to predict net clinical benefit. It may be preferable for future investigators to perform full microsimulation analyses, rather than relying on the ratios we have here derived. Investigators performing microsimulations would have access to the methods and cohort sizes we have used here, obviating the need for ratios like these. Second, we have used literature-derived risks, and our derivation required some assumptions (such as omitting epidural hemorrhages and assuming that survivors of ICH have similar long-term mortality impact as survivors of ischemic stroke, conditioned on disability). Those assumptions may not hold true if interrogated by large future datasets. Third, our meta-model explains a small degree of overall variance (R^2^ = 0.14), and age is an imperfect proxy for life expectancy. Patients whose life expectancy differ considerably from what would be expected from their age (e.g., young patients with many comorbidities or very spry older adults) may not be accurately represented in our analysis. More generally, patients whose risk factor profiles are very different from our synthetic population may have systematic differences from what we have considered. Further, our baseline life expectancy assumption is based on United States life tables, while patients with atrial fibrillation likely have higher age-specific mortality. Life tables specific to the US population with atrial fibrillation, if available, would refine our predictions. And finally, the decision we have here sought to inform–anticoagulation with warfarin–is only one of a number of treatment options available.

Nonetheless, we believe our analysis has important implications. First, decision analyses that have used fixed weighting ratios should be reconsidered in light of the biases that this method has introduced. If refinements to the weighting ratio lead to different recommendations, it may be necessary to revise guidelines based on those analyses. This is most likely to be meaningful for patients at relatively low risk of ischemic stroke. Second, future decision analyses should incorporate weighting ratios that vary with important predictors or downstream morbidity and mortality. For anticoagulation among patients with atrial fibrillation, we have presented such ratios. Third, decision analyses that do not incorporate full simulations should take care not to introduce bias based on life expectancy for events whose implications are lifelong. And finally, to allow decision analysis without microsimulation, efforts to more accurately predict life expectancy should be pursued.

## Conclusion

In sum, we have derived a ratio of QALYs lost to ICH compared with QALYs lost to ischemic stroke among patients with nonvalvular atrial fibrillation, for use in decision-analytic models. If adopted, we expect that this method will reduce age-based bias that has been introduced by use of a fixed weighting ratio, while also improving decision analyses that do not incorporate full microsimulation models.

## Supporting information

S1 Appendix Supplemental material. (PDF)

S2 Appendix Stata code for analysis. (PDF)

## Abbreviations

ATRIA: Anticoagulation and Risk Factors in Atrial Fibrillation cohort study
GWTG: Get With the Guidelines-Stroke
ICH: Intracranial hemorrhage
mRS: Modified Rankin Score
NIHSS: National Institutes of Health Stroke Scale
NINDS: National Institute of Neurological Disorders and Stroke’s (NINDS)
NIS: National Inpatient Sample
NHANES: National Health and Nutrition Examination Survey
RE-LY: Randomized Evaluation of Long-Term Anticoagulation Therapy trial
ROCKET-AF: Rivaroxaban Once Daily Oral Direct Factor Xa Inhibition Compared with Vitamin K Antagonism for Prevention of Stroke and Embolism Trial in Atrial Fibrillation trial
tPA: Tissue plasminogen activator
QALYs: Quality-adjusted life-years
