# Supplementary material for: Reducing age bias in decision analyses of anticoagulation for patients with nonvalvular atrial fibrillation – A microsimulation study
Source: PLoS One. 2018 Jul 11;13(7):e0199593. doi: 10.1371/journal.pone.0199593 (PMC6040745; doi:10.1371/journal.pone.0199593)
Supplement: S1 Appendix — (PDF) [file pone.0199593.s001.pdf]

S1 Appendix. Supplemental material for: Reducing age bias in decision analyses of anticoagulation for patients with atrial fibrillation

Matt Pappas, MD, MPH; Sandeep Vijan, MD, MS; Michael Rothberg, MD, MPH; Daniel Singer, MD, MA

1. Synthetic population 3
2. Step-wise description of analysis steps 3
   A. Initialization/setup 3
   B. Ischemic stroke condition 3
   C. Intracranial hemorrhage condition 4
   D. Comparison between intracranial hemorrhage and ischemic stroke 5
3. Future disability following ischemic stroke 5

1. Synthetic population

A synthetic population is an essential part of this analysis, because hospital mortality has been shown to vary based on the presence of risk factors following either ischemic stroke or intracranial hemorrhage. Unfortunately, atrial fibrillation is not included in NHANES, which is otherwise the most robust nationwide sample of underlying risk factors. We used continuous NHANES data from 2011-2012, assuming all patients over age 80 had risk factor profiles identical to NHANES' "80 and older" group. We sampled using a bootstrapping approach, stratified by age and sex, to create a synthetic U.S. population mirroring the risk factors of the population. We then applied age- and sex-specific prevalence of atrial fibrillation (from the ATRIA cohort) to that population and, for the purposes of this analysis, kept only patients with atrial fibrillation. The most recent NHANES survey that included ankle-brachial index (ABI) was 2003-2004. We therefore created a logistic regression to predict peripheral arterial disease (defined as either ABI greater than 1.4 or less than 0.9), and predicted that diagnosis in our synthetic population.

A sample (due to file size) of our synthetic population is available on request from the corresponding author.

2. Step-wise description of analysis steps

To enhance reproducibility, we here describe our simulation in a step-wise fashion. Origins of point estimates and ranges are reported in the body of the paper, along with a narrative description of our analysis and a schematic of the model. Commented code for analysis is included separately.

A. Initialization/setup

- The synthetic population described above is loaded.
- The probability of peripheral arterial disease is predicted, using earlier years of NHANES data. Predicted probability is dichotomized proportionally at random.
- Stroke risk scores are calculated for each patient, using either CHADS2 or CHADS2-Vasc.
- A baseline modified Rankin Score of 0 is assumed for all patients.
- An identical baseline utility and an additive model of disutility is assumed for all patients.

B. Ischemic stroke condition

- NIHSS is sampled from a normal distribution according to published summary statistics. Non-sensical values (less than zero or greater than 42) are replaced by repeated draws from the same distribution.
- In-hospital mortality is predicted using patient variables, NIHSS, and a previously-published logistic regression model from GWTG-Stroke.
- Patients who took an ambulance from the scene are assumed to be an independent and randomly distributed 53.4% of the population.
- Patients who did not present via the ED are assumed to be an independent and randomly distributed 5.7% of the population.
- Patients who arrived during regular business hours are assumed to be an independent and randomly distributed 46.8% of the population.
- Patients receiving thrombolytics are assumed to be a randomly distributed 10% of the population.
- Inpatient death is dichotomized proportionally at random.
- Modified Rankin score 3 months following the ischemic stroke is predicted using an ordinal logistic regression derived from NINDS data (see below). A weighted average mRS is calculated and

rounded. This represents each patient's "long-term" disability. We assumed no change following 3-month disability.

- Length of stay is sampled from NIS, matched to primary diagnosis group (Ischemic stroke without thrombolytics or Ischemic stroke with thrombolytics)
- We calculated disutility of hospitalization using a method similar to Chit and colleagues, using data gathered by McPhail and colleagues. We used Chit and colleagues' estimates of the disutility of the first and last day of hospitalization, and assumed a linear interpolation between. This is algebraically identical to assuming that the first day of hospitalization confers a disutility of 0.4272, while each subsequent day of hospitalization confers a disutility of 0.288.
- Patients with a length of stay of 0 days are rounded up to a length of stay of 1 day for the purposes of estimating disutility.
- Quality-adjusted life-days during the index hospitalization are discounted to day 0, using standard exponential discounting, and converted to quality-adjusted life-years.
- Post-event life expectancy is calculated, conditional upon age, sex, and modified Rankin score, and using hazard ratios applied to United States Life Tables.
- Long-term disutilities are applied to each patient, conditional on mRS.
- Post-discharge quality-adjusted life-years are discounted to day 0, using standard exponential compounding.

C. Intracranial hemorrhage condition

- Each patient is assigned to sustain an intracerebral, subarachnoid, or subdural hemorrhage proportionally at random.
- For patients who have sustained intracerebral or subarachnoid hemorrhages, NIHSS is drawn from appropriate gamma distributions. Non-sensical values (less than zero or greater than 42) are replaced by repeated draws from the same distribution.
- In-hospital mortality is predicted using patient variables, NIHSS, and a previously-published logistic regression model from GWTG-Stroke.
- All patients are assumed to have been on warfarin and to have a coagulopathy (due to warfarin).
- Intracerebral hemorrhage patients who took an ambulance from the scene are assumed to be an independent and randomly distributed 65.9% of that population.
- Intracerebral hemorrhage patients who did not present via the ED are assumed to be an independent and randomly distributed 7.6% of that population.
- Intracerebral hemorrhage patients who arrived during regular business hours are assumed to be an independent and randomly distributed 40.8% of that population.
- Subarachnoid hemorrhage patients who took an ambulance from the scene are assumed to be an independent and randomly distributed 53.7% of that population.
- Subarachnoid hemorrhage patients who did not present via the ED are assumed to be an independent and randomly distributed 17.1% of that population.
- Subarachnoid hemorrhage patients who arrived during regular business hours are assumed to be an independent and randomly distributed 33.1% of that population.
- We calculated the probability of in-hospital mortality for subdural hemorrhage patients using relevant patient variables and a previously-published multifactorial analysis.
- Inpatient death is dichotomized proportionally at random.
- Conditional on survival to hospital discharge, patients are proportionally assigned long-term modified Rankin scores according to observed probabilities in prior studies (13.8% for each of mRS 0-2, 19.5% for each of mRS 3-5). Probabilities are dichotomized proportionally at random.
- Length of stay is sampled from NIS, matched to primary diagnosis group (Intracerebral hemorrhage, Subarachnoid hemorrhage, and Subdural hemorrhage).

- We calculated disutility of hospitalization using a method similar to Chit and colleagues, using data gathered by McPhail and colleagues. We used Chit and colleagues' estimates of the disutility of the first and last day of hospitalization, and assumed a linear interpolation between. This is algebraically identical to assuming that the first day of hospitalization confers a disutility of 0.4272, while each subsequent day of hospitalization confers a disutility of 0.288.
- Patients with a length of stay of 0 days are rounded up to a length of stay of 1 day for the purposes of estimating disutility.
- Quality-adjusted life days during the index hospitalization are discounted to day 0, using standard exponential discounting, and converted to quality-adjusted life-years.
- Post-event life expectancy is calculated, conditional upon age, sex, and modified Rankin score, and using hazard ratios applied to United States Life Tables.
- Long-term disutilities are applied to each patient, conditional on mRS.
- Post-discharge quality-adjusted life-years are discounted to day 0, using standard exponential compounding.

D. Comparison between intracranial hemorrhage and ischemic stroke

- For each patient, QALYs in the intracranial hemorrhage condition is divided by QALYs in the ischemic stroke condition. This ratio of QALYs lost to ICH relative to QALYs lost to ischemic stroke is our primary outcome and result.
- We construct a meta-model (a standard regression model) to assess how sensitive the output ratio is to patient variables (age, congestive heart failure, hypertension, diabetes, prior stroke, coronary artery disease, dyslipidemia, weight, and discount rate). Coronary artery disease is not a statistically significant predictor of the final ratio, and is therefore dropped from the meta-model. Other predictors are statistically significant.
- We assess whether the ratio is meaningfully sensitive to each dichotomous variable (CHF, hypertension, diabetes, prior stroke, and dyslipidemia). No dichotomous variable leads to a >10% change in the predicted ratio (our definition of an important effect).
- We assess whether the ratio is meaningfully sensitive to each continuous variable (age, weight, and discount rate) when each input parameter is varied from its 5th to 95th percentile value. Weight does not satisfy our definition of an important effect. Varying age from the 5th to 95th percentile led to a 235% change in predicted QALY loss ratio, while varying discount rate led to a 34% change in predicted QALY loss ratio. We conclude that our reported outcome ratio is sensitive to age and discount rate.
- We test for interaction terms between the two remaining variables in our meta-model (age and discount rate), finding an important interaction between the two variables.
- Using the final meta-model, we predict the marginal QALY loss ratio for specified combinations of age and discount rate to demonstrate sensitivity to input conditions.

3. Future disability following ischemic stroke

We performed an ordinal logistic regression on the NINDS-tPA trial data to predict mRS months following discharge, among atrial fibrillation patients who survived to discharge, using NIHSS, age, baseline mRS, and use of t-PA as predictors. Because we expected that the GWTG-Stroke inpatient mortality prediction would be a better-calibrated predictor of death before discharge, we conditioned this regression on survival to discharge. Those interested in reproducing this from the same dataset, using Stata:

```
. ologit rank3m c.baseline i.nrankin c.age i.treatcd if patrial==100 & hdchg!=5

[…]

Ordered logistic regression                          Number of obs   =         93
                                                     LR chi2(7)      =      52.26
                                                     Prob > chi2     =     0.0000
Log likelihood = −146.93877                          Pseudo R2       =     0.1510

------------------------------------------------------------------------------
      rank3m |      Coef.   Std. Err.      z    P>|z|     [95% Conf. Interval]
-------------+----------------------------------------------------------------
    baseline |   .2143078   .0357894     5.99   0.000     .1441619    .2844537
             |
     nrankin |
          1  |   1.921345   .9009003     2.13   0.033     .1556126    3.687077
          2  |   .2743499   1.082268     0.25   0.800    −1.846857    2.395556
          3  |  −.2003374   1.550749    −0.13   0.897    −3.239749    2.839074
          4  |   1.504423   1.517393     0.99   0.321    −1.469613    4.478459
             |
         age |   .0318417   .0221059     1.44   0.150     −.011485    .0751684
   2.treatcd |   .6418311   .4022874     1.60   0.111    −.1466377      1.4303
-------------+----------------------------------------------------------------
       /cut1 |   2.996363    1.69073                     −.3174077    6.310133
       /cut2 |   4.728834   1.726345                       1.34526    8.112408
       /cut3 |     5.1216   1.732927                      1.725125    8.518075
       /cut4 |   6.331271    1.76648                      2.869033    9.793509
       /cut5 |   7.859279   1.825368                      4.281623    11.43693
       /cut6 |    8.86914   1.864487                      5.214812    12.52347
------------------------------------------------------------------------------
```

We assessed the goodness of fit of this model, compared with the original dataset, using both a weighted kappa of the model's weighted average mRS prediction (0.44) and the model's most likely mRS prediction (weighted kappa 0.52).
