# Supplementary material for: Reducing age bias in decision analyses of anticoagulation for patients with nonvalvular atrial fibrillation – A microsimulation study
Source: PLoS One. 2018 Jul 11;13(7):e0199593. doi: 10.1371/journal.pone.0199593 (PMC6040745; doi:10.1371/journal.pone.0199593)
Supplement: S2 Appendix — (PDF) [file pone.0199593.s002.pdf]

```
capture log close                                           /* Close a log if one is open */
cd {path}
cap log using weighting.log,replace                         /* Open a log of this do file */
display "$S_DATE $S_TIME"

*************************************************************************
* Deriving a comparative weight for intracranial hemorrhage compared to *
* thromboembolic stroke                                                  *
* Matt Pappas                                                            *
* Created: August 14, 2015                                               *
* Revised: September 21, 2016                                            *
*************************************************************************

version 13                  /* Set the version of Stata */
set more off                /* Run to the end without pausing */
clear all                   /* Clear anything in memory */
pause on

* Set up parameters and options:
set seed 20150818
scalar stroke_score_to_use = 0                          /* 0=CHADS2 */
scalar annual_discount_rate = 0.03          /* Annual discount rate at which to center (uniform) distribution. */

local path = "{path}"
local data_path = "{data_path}"

quietly do `data_path'update_chads2.ado
quietly do `data_path'update_chads2vasc.ado
quietly do `data_path'update_ischemic_stroke_risk_c2.ado
quietly do `data_path'get_ages_at_death_using_NVSR.ado
quietly do `data_path'get_direct_cost_of_enoxaparin.ado

/* This project aims to derive the appropriate weight to apply to ICH
* compared with ischemic stroke. Singer et al. use 1.5, with sensitivity
* analyses from 1 to 2. Here, using the intermediates of death and
* modified Rankin score, I derive a distribution of that weighting. */

*************************************************************************************
* Part One: Load a synthetic population of A-fib patients, derived from NHANES data *
* using /Users/M/Documents/Synthetic_Population/create_synthetic_population_v03.do  *
*************************************************************************************
use `data_path'synthetic_population.dta, clear
drop afib
*expand 2
gen discount_rate = annual_discount_rate*2*runiform()
gen daily_discount_rate = discount_rate/365.24

* Peripheral artery disease isn't included in later NHANES, and so isn't included in the synthetic population.
Instead, predict it using NHANES 2003-2004, and the variables I used in the synthetic population:
estimates use `path'probability_of_PAD.ster
predict x
gen roll_of_die = runiform()
gen peripheral_artery_disease = (roll_of_die < x)
* Looks like an exponential increase with age, consistent with PAD literature:
*bysort age: egen y = mean(peripheral_artery_disease)
*scatter y age
drop x roll_of_die

merge m:1 age male using `data_path'NVSR_static_life_expectancies.dta
keep if _merge == 3
drop _merge
gen annual_ischemic_stroke_risk = .
gen annual_ICH_risk = .
```

```
gen annual_major_bleeding_risk = .

if stroke_score_to_use == 0 {
    disp "Using CHADS2 score to predict risk of ischemic stroke."
    gen chads2_score = .
    update_chads2
    update_ischemic_stroke_risk_c2
    sum chads2_score,detail
}
else if stroke_score_to_use == 1 {
    disp "Using CHADS2-Vasc score to predict risk of ischemic stroke."
    gen chads2vasc_score = .
    update_chads2_vasc
    update_ischemic_stroke_risk_c2v
    sum chads2vasc_score,detail
}

/* Save this, because STATA doesn't want a smaller/different dataset like the following CSV */
save `data_path'daily_risks.dta, replace

************************************************************************************
* Part Two: Get stroke severity (NIHSS) for each stroke type.                      *
************************************************************************************
* Get stroke severity. Mean and SD are bootstrapped from subset of NINDS trial data with A-fib.
gen intracereb_hem_in_bridge_period = 0
gen subarachnoid_in_bridge_period = 0
gen subdural_hem_in_bridge_period = 0

gen NIHSS_ischemic = .
gen NIHSS_intracerebral = .
gen NIHSS_subarachnoid = .

quietly replace NIHSS_ischemic = rnormal(15.9913,7.297555)
forvalues j = 1/25 {
    quietly replace NIHSS_ischemic = rnormal(15.9913,7.297555) if (NIHSS_ischemic < 0 | NIHSS_ischemic > 42)
}
quietly replace NIHSS_ischemic = round(NIHSS_ischemic)

gen prop_of_ICH_intracereb = 90/(90+8+40)
gen prop_of_ICH_SAH = 8/(90+8+40)
gen prop_of_ICH_SDH = 40/(90+8+40)

gen roll_of_die = runiform()
* First assign the proportion of intracranial hemorrhages that are intracerebral, subarachnoid, or subdural. Among
the warfarin groups in RE-LY and ROCKET-AF (Hankey et al.), there were 261 intracranial hemorrhages; 90 of those
were intracerebral, 8 were subarachnoid, and 40 were subdural. Could add the DOAC groups for larger/more stable
estimates, but DOACs could have a different distribution of ICHs compared with warfarin.
quietly replace intracereb_hem_in_bridge_period = 1 if roll_of_die < prop_of_ICH_intracereb
quietly replace subarachnoid_in_bridge_period = 1 if roll_of_die >= prop_of_ICH_intracereb & roll_of_die <
(prop_of_ICH_intracereb + prop_of_ICH_SAH)
quietly replace subdural_hem_in_bridge_period = 1 if roll_of_die >= (prop_of_ICH_intracereb + prop_of_ICH_SAH)
drop roll_of_die

* Get stroke severity for patients with intracranial hemorrhage and subarachnoid hemorrhage, using NIHSS median/IQR
presented in the Smith 2013 GWTG paper (Amer Heart). Intracerebral hemorrhage could either be normal-ish but does
have skew (median 9, IQR of 3-19). Subarachnoid is highly skewed (3, (0,15)). Below are brute-force gamma
distributions to match the skewed summary statistics; would like to have a more elegant derivation if possible.
* Intracerebral:
quietly replace NIHSS_intracerebral = rgamma(0.825,20) if intracereb_hem_in_bridge_period == 1
forvalues j = 1/25 {
    quietly replace NIHSS_intracerebral = rgamma(0.825,20) if (NIHSS_intracerebral<0 | NIHSS_intracerebral > 42) &
intracereb_hem_in_bridge_period == 1
}
quietly replace NIHSS_intracerebral = round(NIHSS_intracerebral) if intracereb_hem_in_bridge_period == 1
```

```
* Subarachnoid:
quietly replace NIHSS_subarachnoid = rgamma(0.35,45) if subarachnoid_in_bridge_period == 1
forvalues j = 1/25 {
    quietly replace NIHSS_subarachnoid = rgamma(0.35,45) if NIHSS_subarachnoid > 42 & NIHSS_subarachnoid != . &
subarachnoid_in_bridge_period == 1
}
quietly replace NIHSS_subarachnoid = floor(NIHSS_subarachnoid) if subarachnoid_in_bridge_period == 1

*************************************************************************************
* Part Three: Get in-hospital mortality from GWTG scoring system (no beta          *
* coefficients presented in this paper)                                              *
*************************************************************************************

/* Calculate the probability of hospital mortality, according to the logistic regression model presented in Table 4
of Smith's 2010 GWTG paper (Circ, 10/12/2010). */
gen in_hosp_mort_score = 5  /* All patients have A-fib */
* First, add up common variables:
replace in_hosp_mort_score = in_hosp_mort_score + 8 if age >= 80
replace in_hosp_mort_score = in_hosp_mort_score + 6 if age >= 70 & age < 80
replace in_hosp_mort_score = in_hosp_mort_score + 3 if age >= 60 & age < 70

replace in_hosp_mort_score = in_hosp_mort_score + 2 if male == 1
replace in_hosp_mort_score = in_hosp_mort_score + 2 if prior_stroke == 0
replace in_hosp_mort_score = in_hosp_mort_score + 4 if coronary_artery_disease == 1
replace in_hosp_mort_score = in_hosp_mort_score + 3 if dyslipidemia == 0
replace in_hosp_mort_score = in_hosp_mort_score + 3 if peripheral_artery_disease == 1   /* GWTG uses peripheral
vascular disease, whereas I have only PAD. But it should be the largest subset of PVD, and thus a reasonable
approximation. */

* Now add in the rest of the ischemic stroke variables:
gen in_hosp_mort_score_ischemic = in_hosp_mort_score + 1
replace in_hosp_mort_score_ischemic = in_hosp_mort_score_ischemic + 56 if NIHSS_ischemic > 25
replace in_hosp_mort_score_ischemic = in_hosp_mort_score_ischemic + 47 if NIHSS_ischemic >= 21 & NIHSS_ischemic <=
25
replace in_hosp_mort_score_ischemic = in_hosp_mort_score_ischemic + 40 if NIHSS_ischemic >= 16 & NIHSS_ischemic <=
20
replace in_hosp_mort_score_ischemic = in_hosp_mort_score_ischemic + 30 if NIHSS_ischemic >= 11 & NIHSS_ischemic <=
15
replace in_hosp_mort_score_ischemic = in_hosp_mort_score_ischemic + 18 if NIHSS_ischemic >= 6 & NIHSS_ischemic <= 10
replace in_hosp_mort_score_ischemic = in_hosp_mort_score_ischemic + 9 if NIHSS_ischemic >= 3 & NIHSS_ischemic <= 5

gen roll_of_die = runiform()
gen arrived_during_bus_hours_stroke = (roll_of_die < 0.468) /* 46.8% of ischemic stroke patients arrived during
business hours. */
replace in_hosp_mort_score_ischemic = in_hosp_mort_score_ischemic + 1 if arrived_during_bus_hours_stroke == 0

replace roll_of_die = runiform()
gen did_not_present_via_ED_stroke = (roll_of_die < 0.057)
replace in_hosp_mort_score_ischemic = in_hosp_mort_score_ischemic + 8 if did_not_present_via_ED_stroke == 1

replace roll_of_die = runiform()
gen ambulance_from_scene_stroke = (roll_of_die < 0.534)
replace in_hosp_mort_score_ischemic = in_hosp_mort_score_ischemic + 9 if ambulance_from_scene_stroke == 1
drop roll_of_die

* Now add in the rest of the intracerebral hemorrhage variables:
gen in_hosp_mort_score_intracerebral = in_hosp_mort_score + 18
replace in_hosp_mort_score_intracerebral = in_hosp_mort_score_intracerebral + 56 if NIHSS_intracerebral > 25 &
NIHSS_intracerebral != .
replace in_hosp_mort_score_intracerebral = in_hosp_mort_score_intracerebral + 47 if NIHSS_intracerebral >= 21 &
NIHSS_intracerebral <= 25
replace in_hosp_mort_score_intracerebral = in_hosp_mort_score_intracerebral + 40 if NIHSS_intracerebral >= 16 &
NIHSS_intracerebral <= 20
```

```
replace in_hosp_mort_score_intracerebral = in_hosp_mort_score_intracerebral + 30 if NIHSS_intracerebral >= 11 &
NIHSS_intracerebral <= 15
replace in_hosp_mort_score_intracerebral = in_hosp_mort_score_intracerebral + 18 if NIHSS_intracerebral >= 6 &
NIHSS_intracerebral <= 10
replace in_hosp_mort_score_intracerebral = in_hosp_mort_score_intracerebral + 9 if NIHSS_intracerebral >= 3 &
NIHSS_intracerebral <= 5

gen roll_of_die = runiform()
gen arrived_during_bus_hours_intrace = (roll_of_die < 0.408)
replace in_hosp_mort_score_intracerebral = in_hosp_mort_score_intracerebral + 1 if arrived_during_bus_hours_intrace
== 0

replace roll_of_die = runiform()
gen did_not_present_via_ED_intrace = (roll_of_die < 0.076)
replace in_hosp_mort_score_intracerebral = in_hosp_mort_score_intracerebral + 8 if did_not_present_via_ED_intrace
== 1

replace roll_of_die = runiform()
gen ambulance_from_scene_intrace = (roll_of_die < 0.659)
replace in_hosp_mort_score_intracerebral = in_hosp_mort_score_intracerebral + 9 if ambulance_from_scene_intrace == 1
drop roll_of_die

* And the rest of the SAH variables:
gen in_hosp_mort_score_subarachnoid = in_hosp_mort_score + 27
replace in_hosp_mort_score_subarachnoid = in_hosp_mort_score_subarachnoid + 56 if NIHSS_subarachnoid > 25 &
NIHSS_subarachnoid != .
replace in_hosp_mort_score_subarachnoid = in_hosp_mort_score_subarachnoid + 47 if NIHSS_subarachnoid >= 21 &
NIHSS_subarachnoid <= 25
replace in_hosp_mort_score_subarachnoid = in_hosp_mort_score_subarachnoid + 40 if NIHSS_subarachnoid >= 16 &
NIHSS_subarachnoid <= 20
replace in_hosp_mort_score_subarachnoid = in_hosp_mort_score_subarachnoid + 30 if NIHSS_subarachnoid >= 11 &
NIHSS_subarachnoid <= 15
replace in_hosp_mort_score_subarachnoid = in_hosp_mort_score_subarachnoid + 18 if NIHSS_subarachnoid >= 6 &
NIHSS_subarachnoid <= 10
replace in_hosp_mort_score_subarachnoid = in_hosp_mort_score_subarachnoid + 9 if NIHSS_subarachnoid >= 3 &
NIHSS_subarachnoid <= 5

gen roll_of_die = runiform()
gen arrived_during_bus_hours_subara = (roll_of_die < 0.331)
replace in_hosp_mort_score_subarachnoid = in_hosp_mort_score_subarachnoid + 1 if arrived_during_bus_hours_subara ==
0

replace roll_of_die = runiform()
gen did_not_present_via_ED_subara = (roll_of_die < 0.171)
replace in_hosp_mort_score_subarachnoid = in_hosp_mort_score_subarachnoid + 8 if did_not_present_via_ED_subara == 1

replace roll_of_die = runiform()
gen ambulance_from_scene_subara = (roll_of_die < 0.537)
replace in_hosp_mort_score_subarachnoid = in_hosp_mort_score_subarachnoid + 9 if ambulance_from_scene_subara == 1
drop roll_of_die

gen prob_hosp_mortality_ischemic = 1/(1+exp(5.949803-0.066087*in_hosp_mort_score_ischemic))
gen prob_hosp_mortality_intracerebra = 1/(1+exp(5.949803-0.066087*in_hosp_mort_score_intracerebral))
gen prob_hosp_mortality_subarachnoid = 1/(1+exp(5.949803-0.066087*in_hosp_mort_score_subarachnoid))

* Estimate in-hospital mortality from subdural hemorrhages, using the Busl and Prabhakaran article's multifactorial
analysis:
gen prob_hosp_mortality_subdural = 0.118
gen OR_for_SDH_inpt_mort_age = rnormal(1.017,(1.022-1.012)/3.92)
gen OR_for_SDH_inpt_mort_CHF = rnormal(1.42,(1.71-1.19)/3.92)
gen OR_for_SDH_inpt_mort_warf = rnormal(1.41,(1.7-1.17)/3.92)
gen OR_for_SDH_inpt_mort_coag = rnormal(2.14,(2.61-1.75)/3.92)
forvalues i = 1/5 {
    replace OR_for_SDH_inpt_mort_age = rnormal(1.017,(1.022-1.012)/3.92) if OR_for_SDH_inpt_mort_age < 1.012 |
```

```
OR_for_SDH_inpt_mort_age > 1.022
    replace OR_for_SDH_inpt_mort_CHF = rnormal(1.42,(1.71-1.19)/3.92) if OR_for_SDH_inpt_mort_CHF < 1.19 |
OR_for_SDH_inpt_mort_CHF > 1.71
    replace OR_for_SDH_inpt_mort_warf = rnormal(1.41,(1.7-1.17)/3.92) if OR_for_SDH_inpt_mort_warf < 1.17 |
OR_for_SDH_inpt_mort_warf > 1.7
    replace OR_for_SDH_inpt_mort_coag = rnormal(2.14,(2.61-1.75)/3.92) if OR_for_SDH_inpt_mort_coag < 1.75 |
OR_for_SDH_inpt_mort_coag > 2.61
}
* Assume everyone here is on anticoagulation (because that's the decision under consideration) and has a
coagulopathy (because of warfarin):
gen OR_for_SDH_inpt_mort = (OR_for_SDH_inpt_mort_age*age-age)*OR_for_SDH_inpt_mort_warf*OR_for_SDH_inpt_mort_coag
replace OR_for_SDH_inpt_mort = OR_for_SDH_inpt_mort*OR_for_SDH_inpt_mort_CHF if congestive_heart_failure == 1

gen odds_inpt_mort_SDH = prob_hosp_mortality_subdural/(1-prob_hosp_mortality_subdural)
replace odds_inpt_mort_SDH = odds_inpt_mort_SDH*OR_for_SDH_inpt_mort
replace prob_hosp_mortality_subdural = odds_inpt_mort_SDH/(odds_inpt_mort_SDH+1)
drop OR_for_SDH_inpt_mort_age OR_for_SDH_inpt_mort_CHF OR_for_SDH_inpt_mort_warf OR_for_SDH_inpt_mort_coag
OR_for_SDH_inpt_mort odds_inpt_mort_SDH

*************************************************************************************
* Part four: Dichotomize probabilities of death into inpatient death or survival    *
* to discharge. For extracranial major hemorrhage, begin with a static 9.5%         *
* inpatient mortality, from Guerrouij et al.:                                       *
*************************************************************************************
gen died_in_hospital_ischemic = 0
gen died_in_hospital_ICH = 0

* Replace random numbers in between each comparison:
gen roll_of_die = runiform()
quietly replace died_in_hospital_ischemic = 1 if roll_of_die < prob_hosp_mortality_ischemic

quietly replace roll_of_die = runiform()
quietly replace died_in_hospital_ICH = 1 if intracereb_hem_in_bridge_period == 1 & roll_of_die <
prob_hosp_mortality_intracerebra

quietly replace roll_of_die = runiform()
quietly replace died_in_hospital_ICH = 1 if subarachnoid_in_bridge_period == 1 & roll_of_die <
prob_hosp_mortality_subarachnoid

quietly replace roll_of_die = runiform()
quietly replace died_in_hospital_ICH = 1 if subdural_hem_in_bridge_period == 1 & roll_of_die <
prob_hosp_mortality_subdural

drop roll_of_die
pause

*************************************************************************************
* Part Four: Predict modified Rankin scores 3-months following event.               *
*************************************************************************************

/* First, ischemic strokes. Setup for mRS prediction: "baseline" is NIHSS at presentation, "nrankin" is pre-stroke
mRS (here assumed to be 0), and "treatcd" is whether the patient received tPA. This score prediction is contingent
upon survival to hospital discharge. */
gen baseline = NIHSS_ischemic
gen nrankin = 0
gen treatcd = 0
quietly replace treatcd = 1 if runiform()<=0.1  /* This could be improved if I knew how to predict who gets
thrombolytics. Here, I assume that a random 10% of the population gets them.    */
estimates use `data_path'NINDS_mRS_prediction.ster
predict p0 p1 p2 p3 p4 p5 p6
* As a weighted average:
gen predicted_rankin_ischemic = 1*p1 + 2*p2 + 3*p3 + 4*p4 + 5*p5 + 6*p6
quietly replace predicted_rankin_ischemic = round(predicted_rankin_ischemic)
```

```
/* Second, intracranial hemorrhages. Here follow the rates of disability reported by Fang (2007, Am J Med). Pretty
easy to say that no disability = mRS of 0, and inpatient death is taken care of above, so the questions remain of
what to do with minor disability (16%) and major disability (34%). Here, I'm going to say that minor disability is
evenly distributed between 1 and 2, major disability is evenly distributed between 3, 4, and 5, and mRS of 6 (42%
died prior to discharge) is removed from the denominator. Which leaves:
*  0.08/(1-0.42) with an mRS of 0
*  0.16/((1-0.42)*2) with an mRS of 1
*  0.16/((1-0.42)*2) with an mRS of 2
*  0.34/((1-0.42)*3) with an mRS of 3
*  0.34/((1-0.42)*3) with an mRS of 4
*  0.34/((1-0.42)*3) with an mRS of 5
* These aren't separated by subarachnoid/intracerebral, so will assume that all ICHs lead to equivalent
probabilities of mRS scores: */
gen x = runiform()
gen predicted_rankin_ICH = .
quietly replace predicted_rankin_ICH = 0 if x <= 0.08/(1-0.42)
quietly replace predicted_rankin_ICH = 1 if x > 0.08/(1-0.42) & x <= (0.08+0.16*1/2)/(1-0.42)
quietly replace predicted_rankin_ICH = 2 if x > (0.08+0.16*1/2)/(1-0.42) & x <= (0.08+0.16*2/2)/(1-0.42)
quietly replace predicted_rankin_ICH = 3 if x > (0.08+0.16)/(1-0.42) & x <= (0.08+0.16+0.34*1/3)/(1-0.42)
quietly replace predicted_rankin_ICH = 4 if x > (0.08+0.16+0.34*1/3)/(1-0.42) & x <= (0.08+0.16+0.34*2/3)/(1-0.42)
quietly replace predicted_rankin_ICH = 5 if x > (0.08+0.16+0.34*2/3)/(1-0.42) & x <= (0.08+0.16+0.34*3/3)/(1-0.42)
drop x
tab predicted_rankin_ICH

************************************************************************************
* Part five: While I don't use costs in this abstract, the following files from     *
* an earlier analysis have both costs and length of stay from NIS.                 *
************************************************************************************

* Ischemic stroke, without tPA:
merge 1:1 _n using `data_path'costs_ischemic_stroke_no_tPA.dta
keep if _merge==3
drop _merge

* Ischemic stroke, with tPA:
merge 1:1 _n using `data_path'costs_ischemic_stroke_tPA.dta
keep if _merge==3
drop _merge

* Intracerebral hemorrhage:
merge 1:1 _n using `data_path'costs_intracerebral_hemorrhage.dta
keep if _merge==3
drop _merge

* Subarachnoid hemorrhage:
merge 1:1 _n using `data_path'costs_SAH.dta
keep if _merge==3
drop _merge

* Subdural hemorrhage:
merge 1:1 _n using `data_path'costs_subdural.dta
keep if _merge==3
drop _merge

************************************************************************************
* Part six: Get length of stay and QALY loss due to hospitalization.               *
************************************************************************************
* Baseline utility for patients with atrial fib:
gen utility = 0.988

* For the disutility of hospitalization, I'm using data from McPhail et al., similarly to how Chit et al. used it
in their Lancet ID paper (but assuming that utility changes in a linear fashion from the admission value to the
discharge value over the hospitalization). Then discount each from future value to present value.
gen length_of_stay_ischemic = .
```

```
gen length_of_stay_ICH = .

replace length_of_stay_ICH = LOS_SAH if subarachnoid_in_bridge_period == 1
replace length_of_stay_ICH = LOS_ICH if intracereb_hem_in_bridge_period == 1
replace length_of_stay_ICH = LOS_SDH if subdural_hem_in_bridge_period == 1
replace length_of_stay_ischemic = LOS_stroke_tPA if treatcd==1
replace length_of_stay_ischemic = LOS_stroke_no_tPA if treatcd==0

* Can drop some NIS-imported variables to free up some memory now:
drop LOS_SAH LOS_ICH LOS_SDH LOS_stroke_tPA LOS_stroke_no_tPA cost_hosp_ischemic_stroke_no_tPA
cost_hosp_ischemic_stroke_tPA cost_hosp_intracereb_hem cost_hosp_SAH cost_hosp_subdural p0 p1 p2 p3 p4 p5 p6
LOS_stroke_no_tPA LOS_stroke_tPA LOS_ICH LOS_SAH LOS_SDH

* For patients with an estimated LoS of 0 days, round up to 1 day:
replace length_of_stay_ischemic = 1 if length_of_stay_ischemic == 0
replace length_of_stay_ICH = 1 if length_of_stay_ICH == 0

quietly sum length_of_stay_ischemic
local highest_LOS_ischemic = r(max)
quietly sum length_of_stay_ICH
local highest_LOS_ICH = r(max)

disp `highest_LOS_ischemic'
disp `highest_LOS_ICH'

forvalues i = 1(1)`highest_LOS_ischemic' {
    quietly gen disutil_ischemic_hosp_day_`i' = 0
    quietly replace disutil_ischemic_hosp_day_`i' = -0.1392 if `i' == length_of_stay_ischemic
    quietly replace disutil_ischemic_hosp_day_`i' = -0.4272 if `i' == 1
    quietly replace disutil_ischemic_hosp_day_`i' = (0.4272-0.1392)*(`i'-1)/(length_of_stay_ischemic-1)-0.4272 if
`i' > 1 & `i' < length_of_stay_ischemic
    quietly gen total_util_ischemic_hosp_day_`i' = utility + disutil_ischemic_hosp_day_`i'
}

forvalues i = 1(1)`highest_LOS_ICH' {
    quietly gen disutil_ICH_hosp_day_`i' = 0
    quietly replace disutil_ICH_hosp_day_`i' = -0.1392 if `i' == length_of_stay_ICH
    quietly replace disutil_ICH_hosp_day_`i' = -0.4272 if `i' == 1
    quietly replace disutil_ICH_hosp_day_`i' = (0.4272-0.1392)*(`i'-1)/(length_of_stay_ICH-1)-0.4272 if `i' > 1 &
`i' < length_of_stay_ICH
    quietly gen total_util_ICH_hosp_day_`i' = utility + disutil_ICH_hosp_day_`i'
}

gen discounted_QALYs_ischemic = 0
gen discounted_QALYs_ICH = 0

forvalues i = 1(1)`highest_LOS_ischemic' {
    * First, get discounted QAL days, *not* years:
    quietly replace discounted_QALYs_ischemic = discounted_QALYs_ischemic +
((total_util_ischemic_hosp_day_`i')/(1+daily_discount_rate)^(`i'))
    quietly drop disutil_ischemic_hosp_day_`i' total_util_ischemic_hosp_day_`i'
}
quietly replace discounted_QALYs_ischemic = discounted_QALYs_ischemic/365.24

forvalues i = 1(1)`highest_LOS_ICH' {
    * First, get discounted QAL days, *not* years:
    quietly replace discounted_QALYs_ICH = discounted_QALYs_ICH +
((total_util_ICH_hosp_day_`i')/(1+daily_discount_rate)^(`i'))
    quietly drop disutil_ICH_hosp_day_`i' total_util_ICH_hosp_day_`i'
}
quietly replace discounted_QALYs_ICH = discounted_QALYs_ICH/365.24

*************************************************************************************
* Part eight: Get post-hospitalization QALY loss.                                   *
```

```
********************************************************************************

* (Code to simulate life expectancy for post-event patients deleted. To reduce variance and enhance repeatability,
this code uses a static life expectancy calculated by applying the HRs to life tables.)

* And here I use life-table-calculated life expectancy, rather than the full life span simulation above:
gen post_discharge_LE_ischemic = nvsr_life_expectancy
replace post_discharge_LE_ischemic = nvsr_life_expectancy_mrs02 if predicted_rankin_ischemic <= 2 &
died_in_hospital_ischemic == 0
replace post_discharge_LE_ischemic = nvsr_life_expectancy_mrs34 if (predicted_rankin_ischemic == 3 |
predicted_rankin_ischemic == 4) & died_in_hospital_ischemic == 0
replace post_discharge_LE_ischemic = nvsr_life_expectancy_mrs5 if predicted_rankin_ischemic == 5 &
died_in_hospital_ischemic == 0
replace post_discharge_LE_ischemic = 0 if predicted_rankin_ischemic == 6 & died_in_hospital_ischemic == 0

gen post_discharge_LE_ICH = nvsr_life_expectancy
replace post_discharge_LE_ICH = nvsr_life_expectancy_mrs02 if predicted_rankin_ICH <= 2 & died_in_hospital_ICH == 0
replace post_discharge_LE_ICH = nvsr_life_expectancy_mrs34 if (predicted_rankin_ICH == 3 | predicted_rankin_ICH ==
4) & died_in_hospital_ICH == 0
replace post_discharge_LE_ICH = nvsr_life_expectancy_mrs5 if predicted_rankin_ICH == 5 & died_in_hospital_ICH == 0
replace post_discharge_LE_ICH = 0 if predicted_rankin_ICH == 6 & died_in_hospital_ICH == 0

* Using WHO weights from 2009 Stroke paper by Hong and Saver:
gen postdischarge_QALYs_ischemic = 0 if died_in_hospital_ischemic == 1
gen postdischarge_QALYs_ICH = 0 if died_in_hospital_ICH == 1

* Using WHO weights from 2009 Stroke paper by Hong and Saver, assign utility of remaining life expectancy.
replace postdischarge_QALYs_ischemic = post_discharge_LE_ischemic*(utility-0.046) if predicted_rankin_ischemic <= 1
& died_in_hospital_ischemic == 0
replace postdischarge_QALYs_ischemic = post_discharge_LE_ischemic*(utility-0.212) if predicted_rankin_ischemic == 2
& died_in_hospital_ischemic == 0
replace postdischarge_QALYs_ischemic = post_discharge_LE_ischemic*(utility-0.331) if predicted_rankin_ischemic == 3
& died_in_hospital_ischemic == 0
replace postdischarge_QALYs_ischemic = post_discharge_LE_ischemic*(utility-0.652) if predicted_rankin_ischemic == 4
& died_in_hospital_ischemic == 0
replace postdischarge_QALYs_ischemic = post_discharge_LE_ischemic*(utility-0.944) if predicted_rankin_ischemic == 5
& died_in_hospital_ischemic == 0
replace postdischarge_QALYs_ischemic = 0 if predicted_rankin_ischemic == 6 | died_in_hospital_ischemic == 1

replace postdischarge_QALYs_ICH = post_discharge_LE_ICH*(utility-0.046) if predicted_rankin_ICH <= 1 &
died_in_hospital_ICH == 0
replace postdischarge_QALYs_ICH = post_discharge_LE_ICH*(utility-0.212) if predicted_rankin_ICH == 2 &
died_in_hospital_ICH == 0
replace postdischarge_QALYs_ICH = post_discharge_LE_ICH*(utility-0.331) if predicted_rankin_ICH == 3 &
died_in_hospital_ICH == 0
replace postdischarge_QALYs_ICH = post_discharge_LE_ICH*(utility-0.652) if predicted_rankin_ICH == 4 &
died_in_hospital_ICH == 0
replace postdischarge_QALYs_ICH = post_discharge_LE_ICH*(utility-0.944) if predicted_rankin_ICH == 5 &
died_in_hospital_ICH == 0
replace postdischarge_QALYs_ICH = 0 if predicted_rankin_ICH == 6 | died_in_hospital_ICH == 1

* Discount QALYs to present, using continuous exponential compounding:
replace discounted_QALYs_ischemic = discounted_QALYs_ischemic +
(1-exp(-discount_rate*postdischarge_QALYs_ischemic))/discount_rate
replace discounted_QALYs_ICH = discounted_QALYs_ICH + (1-exp(-discount_rate*postdischarge_QALYs_ICH))/discount_rate

* Now subtract discounted QALYs from discounted baseline expected QALYs, to get QALY loss with ischemic stroke and
ICH:
gen discounted_QALYs_baseline = (1-exp(-discount_rate*nvsr_life_expectancy*utility))/discount_rate
gen QALY_loss_ischemic = discounted_QALYs_baseline - discounted_QALYs_ischemic
gen QALY_loss_ICH = discounted_QALYs_baseline - discounted_QALYs_ICH

********************************************************************************
* Part Nine: Put this all together. Here we're interested in the ratio of QALY loss *
```

```
* in ICH relative to that of ischemic strokes.                                    *
*************************************************************************************

gen ICH_to_ischemic_ratio = QALY_loss_ICH/QALY_loss_ischemic

sum ICH_to_ischemic_ratio,detail
hist ICH_to_ischemic_ratio if ICH_to_ischemic_ratio <=4

* Intermediate results for publication:
sum died_in_hospital_ischemic
sum died_in_hospital_ICH
sum died_in_hospital_ICH if intracereb_hem_in_bridge_period == 1
sum died_in_hospital_ICH if subarachnoid_in_bridge_period == 1
sum died_in_hospital_ICH if subdural_hem_in_bridge_period == 1

sum predicted_rankin_ischemic,detail
sum predicted_rankin_ICH,detail
sum predicted_rankin_ICH if intracereb_hem_in_bridge_period == 1
sum predicted_rankin_ICH if subarachnoid_in_bridge_period == 1
sum predicted_rankin_ICH if subdural_hem_in_bridge_period == 1

tab predicted_rankin_ischemic
tab predicted_rankin_ICH

*************************************************************************************
* Part Nine: Meta-model!                                                            *
*************************************************************************************
regress ICH_to_ischemic_ratio c.age i.congestive_heart_failure i.hypertension i.diabetes i.prior_stroke
i.coronary_artery_disease i.dyslipidemia c.weight
* Only CAD is not statistically significant. Can remove that.
regress ICH_to_ischemic_ratio c.age i.congestive_heart_failure i.hypertension i.diabetes i.prior_stroke
i.dyslipidemia c.weight c.discount_rate

* Dichotomous variables:
margins, at(congestive_heart_failure = (0 1))
disp el(r(b),1,2)/el(r(b),1,1)

margins, at(hypertension = (0 1))
disp el(r(b),1,2)/el(r(b),1,1)

margins, at(diabetes = (0 1))
disp el(r(b),1,2)/el(r(b),1,1)

margins, at(prior_stroke = (0 1))
disp el(r(b),1,2)/el(r(b),1,1)

margins, at(dyslipidemia = (0 1))
disp el(r(b),1,2)/el(r(b),1,1)

* Continuous variables:
sum weight,detail
scalar lower_bound = r(p5)
scalar upper_bound = r(p95)
margins, at(weight = (`=lower_bound' `=upper_bound'))
disp el(r(b),1,2)/el(r(b),1,1)

sum age,detail
scalar lower_bound = r(p5)
scalar upper_bound = r(p95)
margins, at(age = (`=lower_bound' `=upper_bound'))
disp el(r(b),1,2)/el(r(b),1,1)

sum discount_rate,detail
scalar lower_bound = r(p5)
```

```
scalar upper_bound = r(p95)
margins, at(discount_rate = (`=lower_bound' `=upper_bound'))
disp el(r(b),1,2)/el(r(b),1,1)

* None of the variables other than age and discount rate had a >10% impact on the predicted ratio; all the others can be pared.

* Test for higher-order polynomial effects, and compare the impact over the range of age and discount rate:
regress ICH_to_ischemic_ratio c.age##c.age c.discount_rate
sum age,detail
scalar lower_bound = r(p5)
scalar upper_bound = r(p95)
margins, at(age = (`=lower_bound' `=upper_bound'))
* From age 45 to 91, the range is 2.75 (at age 45) to 1.17 (at age 91). Now without the interaction:
regress ICH_to_ischemic_ratio c.age
sum age,detail
scalar lower_bound = r(p5)
scalar upper_bound = r(p95)
margins, at(age = (`=lower_bound' `=upper_bound'))
* Range is now from 2.62 (at age 45) to 0.85 (at age 91). So there's a small effect at lower ages, but a greater effect at older ages (~38% difference at age 91). Will keep interaction term.

regress ICH_to_ischemic_ratio c.discount_rate##c.discount_rate c.age##c.age
sum discount_rate,detail
scalar lower_bound = r(p5)
scalar upper_bound = r(p95)
margins, at(discount_rate = (`=lower_bound' `=upper_bound'))
* From discount rate, the range is 1.40 (0.3%) to 1.88 (5.7%). Now without the interaction:
regress ICH_to_ischemic_ratio c.discount_rate c.age##c.age
sum discount_rate,detail
scalar lower_bound = r(p5)
scalar upper_bound = r(p95)
margins, at(discount_rate = (`=lower_bound' `=upper_bound'))
* Range is now from 1.35 (0.3%) to 1.83 (5.7%). So there seems to be a small but consistent effect. Now try interaction with age:
regress ICH_to_ischemic_ratio c.discount_rate##c.age c.age##c.age
sum discount_rate,detail
scalar lower_bound = r(p5)
scalar upper_bound = r(p95)
margins, at(discount_rate = (`=lower_bound' `=upper_bound'))
* Range is now 1.35 to 1.83. So same average effect at either end of the range as the second-order interaction. But on marginsplot, the plots cross just after age 80. That crossing goes away with third-order interactions, so I think it's likely not real.

*Final model and Figure 2:
regress ICH_to_ischemic_ratio c.age##c.age c.discount_rate##c.discount_rate
margins, at(age = (40(1)90) discount_rate=(0(0.01) 0.06))
marginsplot

* For Table 3:
margins, at(age = (40) discount_rate=(0.02 0.04 0.06))
margins, at(age = (50) discount_rate=(0.02 0.04 0.06))
margins, at(age = (60) discount_rate=(0.02 0.04 0.06))
margins, at(age = (70) discount_rate=(0.02 0.04 0.06))
margins, at(age = (80) discount_rate=(0.02 0.04 0.06))
margins, at(age = (90) discount_rate=(0.02 0.04 0.06))

sum ICH_to_ischemic_ratio,detail
/*twoway (histogram ICH_to_ischemic_ratio if ICH_to_ischemic_ratio<=5 & age<=40, color(blue)) ///
       (histogram ICH_to_ischemic_ratio if ICH_to_ischemic_ratio<=5 & age>=90, color(green))
*/

log close
```
